# James Greig Smith

**Published:** 1897-06

**Authors:** 


The Bristol

Medico-Chirurgical Journal.

JUNE, 1897.

### In Memoriam.

### JAMES GREIG SMITH,

M.A., M.B., C.M. Aberd., F.R.S.E.

At the funeral service of James Greig Smith, on June 1st, in the Church at Redland Green, the presence of a large number of mourners, including doctors, students, nurses, and personal friends, gave eloquent testimony to the widespread feeling of regret that, at the early age of 43, his life-work should have come almost suddenly to an end. His death had been the one topic of conversation amongst all ranks in and around the city, for he had made himself a reputation which had placed him in the front rank of his profession, and his work had brought him into contact with a very large circle of friends and patients. The illness which led to the fatal end was so short that the news of his death was a terrible shock to many who had not heard even that he was ill, and this, added to the sense of loss which it was felt that the neighbourhood had sustained, intensified the grief which was manifested on all sides.

Although apparently possessed of much physical vigour,

Greig Smith was by no means a man of strong bodily health. He suffered frequently from various developments of the rheumatic diathesis, having had acute articular rheumatism badly at least twice. Three years ago he had an attack of pneumonia which impaired his strength for some weeks, and perhaps, to a slight degree, permanently. On the morning of Monday, May 24th, he felt some pain in the left side of the chest, but attached no importance to it, thinking it rheumatism of the intercostal muscles. During the day he went to a consultation in the neighbourhood of Swindon, having for part of the journey to drive in an open carriage. In the evening, after his return to Clifton, he formed one of a dinner-party of congenial friends, to one of whom he remarked that he thought he had mischief in his left lung. After dinner he seemed bright, but left early. In the course of the night he vomited, and had a rigor and a temperature of 103°. Early in the morning he was seen by Dr. James Swain, who heard pleuritic rubbing in the left infra-axillary area. Some few hours later, when Dr. Shaw and Dr. Swain saw him together, there was distinct impairment of resonance over the lower lobe of the left lung from the posterior border to the posterior axillary line. The pulse was extremely small and rapid, the dyspnœa great, and the frequent cough brought away clear sputum brightly tinged with blood. On Tuesday night he had very little sleep, but on Wednesday morning he seemed rather better and did not seem to lose ground through the day. In the early part of Thursday he was doing fairly well, but about noon he became somewhat suddenly worse, complaining of pain in the head, and he was extremely restless. At 3 p.m. the signs of lung consolidation were found to have increased considerably. During the next few hours he became dimmed in consciousness, and in the course of the evening Cheyne-Stokes breathing was gradually developed. At 11 p.m. he was barely conscious. Through the night he became steadily worse, and he died at ten minutes past eight on the morning of Friday, the 28th.

Now that his place is vacant we are left to ponder over his life's work, and try to learn the lessons which may be derived from his short but eminently useful career. That it has been exceptionally useful all will gladly admit, for he was a pioneer in many things, and those who were much associated with him have profited greatly by his example and have been roused by his enthusiasm to a higher level of professional work. It may be truly said of him that he left the world a little better than he found it.

James Greig Smith, the son of Mr. Duncan Greig Smith, was born at Nigg, a village a few miles from Aberdeen, on June 21st, 1854. He received his early education at the Aberdeen Grammar School, a foundation of greater antiquity than the celebrated University of that city. Placed, on his entry in 1865, in the lowest or first class by the then rector, Mr. William Barrack, he passed up the classes year by year until he reached the fifth or highest in 1869. The numbers of the school at the time of Mr. Barrack's rectorship show in what reputation it was held as an educational centre, for in 1865 there were 328 boys there. There are unfortunately no records in existence of the prizes or distinctions gained by the boys, but in 1869 on leaving school Greig Smith's name appears sixth in a list which includes forty names placed in order of merit.

Leaving school, Greig Smith entered the University as an Arts Student in the winter session of 1869–70, gaining by open competition a bursary of £14 a year. During the time he studied the Arts he gained prizes in English, Logic, and Natural History, and in March, 1873, he graduated M.A., taking Honours in Natural Science. In his medical studies, his career was again marked with distinction, for he took the second prize in Botany, and the first in Surgery, Practice of Medicine, and Medical Jurisprudence, and finally graduated M.B., C.M., "with honourable distinction" in 1876. While a student he was clinical clerk to the then professor of surgery, Professor Pirrie, at the Aberdeen Royal Infirmary, and University Assistant to him, 1874–5. A year later saw him House Surgeon at that institution.

With taking his degree his association with his University did not cease, for in October, 1881, he was appointed external or extra-professorial examiner in surgery and midwifery; but in April, 1883, on the ground of "the work being heavier than he expected, and occupying more time than he could spare," he requested the University Court to accept his resignation—a somewhat unusual course, but one to which the Court gave its assent. It need hardly be said that his services were much appreciated, but we have it on the authority of one who knew him well that "his dignity as an examiner was sometimes imperilled by his sense of humour."

Always pleased to honour its fellow-countrymen, the Royal Society of Edinburgh, on March 5, 1883, elected him a Fellow, when his name was proposed by Professor Stirling, Dr. Ogilvie Will, Professor Lister, and Dr. Henry Marshall. In the same year he married Miss Adela Ashby, who, with their only child, a daughter, survives him.

After his short experience as resident at Aberdeen, Greig Smith was appointed, in June, 1876, Assistant House Surgeon to the Bristol Royal Infirmary, the patients of which had been moved in the previous September to premises in Colston Street, which were used as a temporary hospital till October, 1876, while the Infirmary was undergoing sanitary renovation.

The Infirmary issued in 1879 its first and only volume of Reports. Of this, Greig Smith, who, after filling the posts of House Surgeon and Medical Superintendent, had then become Junior Surgeon to the Institution, was the Surgical Editor. He contributed to it an elaborate paper on "The Pathology and Treatment of Chronic Osteo-arthritis," in which he dealt specially with the natural history of the pathological processes which lead up to the disease. This article, which was embellished with ten plates lithographed from his own drawings, is marked by that care of observation and literary finish which were prominent features in all his after-work. It is also an excellent example of the endeavour which he made throughout the whole of his surgical career to constitute his practice a legitimate deduction from pathology. The volume contained some other noteworthy contributions from him, including an exhaustive surgical report, which, unfinished by Mr. R. W. Tibbits at the time of his death, was completed by Greig Smith with that analytical ability for which he was so greatly distinguished. In the three years after the re-occupation of the Infirmary, *i.e.* up to the time of the issue of the Reports, there were no deaths from pyæmia or erysipelas, although those diseases had claimed forty-three deaths in the two years and eight and a-half months prior to the closing of the Institution. Notwithstanding this immunity,—which was not considered to be entirely due to the antiseptic method of Lister then in vogue,—the general death-rate was higher, and Mr. Greig Smith in this report dealt in a masterly way with the facts which show that a high hospital-mortality is no evidence in itself of defective sanitation.

Greig Smith, soon after his coming to Bristol, was an active member of the Society under whose auspices this *Journal* is issued. In 1878 the Bristol Medico-Chirurgical Society published a first volume of Transactions, including within the range of its record much of the work done from 1874 to 1878. The volume contains two contributions from Greig Smith. The first was a communication which he made to the Society in October, 1877, on the evening of his election, dealing with two cases of acute miliary tuberculosis. This was illustrated with microscopic specimens and drawings from his facile pencil, and with some detail he traced anatomically the course of infection in these cases. This paper, and also one on "Hemorrhage into the Pons and Medulla," are both illustrated in the volume of Transactions, which also contains a contribution which he made, in November, 1877, to a debate on "The Treatment of High Temperature of the Body," in which he gave an account of the results of treatment by cold baths and quinine in thirty-two cases in which each had been employed.

From this time onward Greig Smith was a frequent contributor to the work of the Society. In April, 1879, he read in outline the paper "On Osteo-arthritis," which was printed in full in the Infirmary Reports. In September of the same year, he showed a patient on whom he had successfully performed his operation for extroversion of the bladder and epispadias. In January, 1881, he read an important communication "On the Histology of Fracture and the Formation of Callus." This, which, somewhat amplified, was published in the *Journal of Anatomy and Physiology*, he was always accustomed to look back upon as the best work he ever did. It was an admirably-written paper, marked by careful and laborious work done in the laboratory by himself, rather than showing an extensive knowledge of the work done by others in the same department; but it illustrates in this respect his well-known characteristic of self-reliance. He paid very little attention to the opinions of others, unless they were confirmed by his own observations. He relied upon his own work, and was guided entirely by his own experiment and research. In the following March, he gave particulars of two cases in which he had done Bigelow's recently-introduced operation of litholapaxy. Taking as a foundation the case of a patient with multiple sarcomata, he brought forward, in November of the same year, the result of his careful and original investigations on the pathology of these tumours. In February, 1882, he reported a gastrostomy which he had done in a case of epithelioma of the œsophagus.

In January, 1882, the Committee decided to recommend the Society to publish a second volume of Transactions, dealing with the communications of the previous four years; but when, at the meeting in September, it was reported that a satisfactory amount of material was not forthcoming for the purpose, and it was thereupon proposed to postpone the issue of the volume for a time, Greig Smith, who had been elected on the Committee in the previous February, seconded an amendment that an attempt should be made to consolidate the medical and surgical interests of the neighbourhood, with a view to the issuing of a publication which should embrace the work of all the men in the district. This amendment was, however, rejected, for its proposer and its seconder were the only persons who voted for it.

But in this particular matter the Committee did not accurately represent either the views of the Society or even of themselves, for at the general meeting in the following month a resolution, in words almost identical with the rejected amendment, was carried without a dissentient. Greig Smith was again the seconder of the proposed innovation. A sub-committee was requested to report on the question, and to this he was appointed secretary. It was doubtless through the reputation he had gained as Surgical Editor of the Infirmary Reports that he was nominated editor of the contemplated journal, and he sent out a circular to the practitioners of the West of England and South Wales asking for their co-operation. The *raison d'être* of the journal he well expressed in the statement that "Such a publication issued in our midst and supported by ourselves would not only rescue from oblivion much that might otherwise be lost, but would also stimulate us more keenly in our efforts to advance medical science."

The appeal met with a considerable amount of support, and in July, 1883, the first number of *The Bristol Medico-Chirurgical Journal* was issued. Greig Smith put into the new venture all his professional and literary influence, and for it he worked hard; and although he felt bound to relinquish the editorship in 1892, he never ceased to take a cordial interest in its welfare, and from time to time still wrote for it, and in this number containing his obituary notice we have the melancholy privilege of chronicling as a book review some work of his which must have been amongst the latest of his literary doings. During the whole period of its existence it was a satisfaction to him to see the bantling, for the birth of which he was largely responsible, growing in influence year by year, and flourishing with a professional and financial success which has been vouchsafed to few journals of its kind.

During the time Greig Smith was editing the *Journal*, he was very busy in the work of the Society recording cases of operation—mostly in abdominal surgery—showing many specimens, and joining in his own luminous way in the discussions. In March, 1888, he described a case of cholecystotomy in which a stone the size of a walnut had been removed.

In 1888 Mr. Greig Smith was made an Honorary Fellow of the American Association of Obstetricians and Gynecologists, and from 1890 to 1892 he was a Vice-President of the British Gynæcological Society.

He did much work in addition to that for the Bristol Society, and the bibliography which appears at the end of this notice will give some idea of his remarkable industry in advancing the knowledge of the subjects to which he gave his consideration.

Greig Smith became president of the Society in 1893, and took "Modern Medical Journalism" for the subject of his address. He was led to this by his experience as editor of the Society's *Journal*, a post which he had resigned only a short time before. He thought that modern medical journalism was decaying and getting stained by the trail of the advertiser, commercial and professional. If he took too gloomy a view of his subject, judging the whole of literature by some unfortunate examples, and forgetting that medicine and its allied sciences are represented to-day by journals of the highest class, the defects of his position were largely atoned for by the vigour of its presentment. The thought came over him, as he said, that perhaps after all he was in this question only a dreamer of dreams. And certainly he was so when he imagined it possible to re-establish a sort of Star-Chamber of medical literature which would permit no paper to be published unless it had received the approval of someone who might possibly know less of the subject than the author himself. For him Milton's *Areopagitica* had been written in vain. And yet those who desire the best results in medical journalism must to a certain extent sympathise with Greig Smith's lament. He was right in deploring the amount of scribbling done only in self-interest. But as in things spiritual so in things medical, if we gather up the tares we may root up the wheat with them. They must unfortunately grow together. But we can all go a considerable distance with Greig Smith and set our faces sternly against all work done, either in periodical literature or in pseudo-book form, for mere personal gain. And when, in the peroration of his address, he urged his hearers on bringing their work before others to diligently prepare their material, to avoid diffuseness, and to speak out their beliefs boldly and manfully, we know that those were the aims he set before himself, and those which he diligently laboured to attain.

Greig Smith has been taken from us with such startling rapidity that it is difficult to realise that for us there is an end of that marvellous activity, that fertility of resource, that originality of matter and manner which ever characterised him, and far outside the membership of the Bristol Medico-Chirurgical Society will often come the sigh "for the touch of a vanished hand and the sound of a voice that is still," and we would fain believe of him that

> "Somewhere, out of human view,
> Whate'er [his] hands are set to do
> Is wrought with tumult of acclaim."

In 1894 Greig Smith was chosen to deliver the address in Surgery at the meeting of the British Medical Association held in Bristol, and those who were present were charmed by the simplicity and beauty of the language, as well as by the powerful arguments by which he advocated an increased attention to the Art of the Surgeon. This was always one of his favourite topics, and he was himself a forcible example of the advantage to an operator of cultivating the art as well as the science of surgery: for to him was ever present the paramount importance of the combination of hand with head, by which alone can be obtained the necessary qualification for the best surgical work, and though with him the brain-work in devising took the lead of the details in execution, yet care in manual and digital practice was always conspicuous as an essential element of his success.

Greig Smith was very rapid in diagnosis, especially in abdominal cases, and sometimes startled both patient and medical attendant by the readiness with which he gave his opinion on an abdominal case to which he had been called in consultation. He very seldom made a vaginal examination, either in cases of ovarian cyst or of large uterine fibroids, and was accustomed to say that if the case could not be diagnosed by abdominal palpation it could not be diagnosed at all.

In the operation room his nervous energy found its fullest scope. In the performance of a critical operation he seemed sometimes to quiver with excitement. He was accustomed to talk almost incessantly while operating, more to himself than to others, and seemed by this means to find some vent for his feelings. He attributed great importance to a good assistant, and during the performance of an operation he would strongly disapprove of any departure from the instructions he had laid down, and woe betide anyone who, by talking on some other subject or by opening the door during the operation, attracted his displeasure. In the case of any emergency arising in the operation room, such as asphyxia during anæsthesia, his fullest energy was at once called forth. He would brush aside all assistants and invert the patient by his own strong arms, or he would seize the upper extremities and carry on artificial respiration until the crisis was over.

Greig Smith was early attracted to the subject of abdominal surgery, and from the outset of his career made it his especial study. He was a great admirer of Thomas Keith, whom he had seen operate on several occasions, and he took early opportunity to visit the operation rooms of other famous ovariotomists, such as Lawson Tait and Knowsley Thornton, from whom he received much kindness, and of whom he was accustomed to speak with great admiration. He always performed his ovariotomies and other abdominal operations in the general operating theatre, and never made any restrictions in the number of students or medical men present, but only made the request that they should not enter or depart during the actual performance of the operation. Greig Smith had probably performed some 300 ovariotomies, but he never published any exact statistics, although his rate of mortality was certainly very low, and his eminence in this department of work was justly recognised when he was asked to write the article on Ovariotomy in Allbutt and Playfair's recently published *System of Gynæcology*. He was very successful in the operation of complete hysterectomy for fibroids, which he performed with great quickness and dexterity, and which in his hands proved a very safe procedure. Supra-vaginal hysterectomy for malignant disease was also with him a very successful operation, and he hardly ever lost a patient after it. The operation for ruptured perineum was one in which he took great interest; he very early had the opportunity of seeing Lawson Tait perform his operation for this condition, and adopted it at once, although he afterwards made some slight modifications of his own. Of late years intestinal surgery occupied more of his attention than any other branch, and he was unceasing in his efforts to effect improvement in this somewhat unsatisfactory department. He took part in the discussion on "The Treatment of Intestinal Obstruction" at the Annual Meeting of the British Medical Association, held in Birmingham in 1890, and he there advocated a method of treatment by intestinal incision and drainage which he constantly adopted in his own practice; he was accustomed to point out that many an acute case of intestinal obstruction, which will not admit of any extensive operation for its relief, may be tided over the critical period by the temporary relief thus afforded, and he never ceased to advocate this method of dealing with severe cases of this affection in preference to more prolonged and serious operations which the patient could not bear. In the operative treatment of cases of malignant disease of the intestine he early decided on a method which he found to be very successful; he performed the operation in two stages, first bringing the portion of gut involved in the growth outside the abdomen and fixing it there, afterwards, when adhesions had formed, removing the diseased bowel, and leaving an artificial anus to be dealt with at a later date. Many a patient with this hitherto intractable malady did he rescue from death and restore to comparative comfort. In this operation, as well as in the operations of gastrostomy and inguinal colotomy, his success was greatly aided by the principle of suturing the viscus to the raw parietal wound and not to the parietal peritoneum; he had numerous specimens which showed how firmly the stomach or the intestines would adhere, in a few hours, to a raw cut muscular or fibrous surface, whereas the union of peritoneum to peritoneum in the same period would be comparatively slight. His operations of gastrostomy, colotomy, and enterostomy were always performed in this way, and he attributed much of his success to this cause.

The work by which the name of Greig Smith will be best perpetuated is of course his book on Abdominal Surgery, which was his *magnum opus*, and the bringing out of which was to him a veritable labour of love. This is neither the time nor the place to review this work; suffice it to say that it has already run through five editions, and he was engaged in a sixth edition at the time of his death. The book filled a void much felt at the time of its first publication, and was welcomed in the warmest terms not only in this country and America, but also on the Continent. The fame of the book constantly brought him friendly communications and criticisms from ali parts of the world, and these, which were much valued by him, were always turned to account in subsequent editions.

In 1888 Greig Smith became Lecturer on Surgery in University College, Bristol, the Chair of which was afterwards made Professorial. To this congenial work he devoted himself with that ardour which was so characteristic of him. In addition to lecturing during the whole of the winter session, he spent much time in the preparation and mounting of specimens, as well as drawings, to illustrate his lectures, and improve the museum of the Medical School. In this labour he was indefatigable, and the museum has been enriched by a large number of valuable specimens prepared entirely by his own hands, and which will remain as a lasting memorial of his enthusiasm and industry. As a teacher, he was forcible, lucid, and thorough. He was an excellent draughtsman, and was fond of illustrating everything he taught by a drawing. His earnestness and obvious love of his subject always proved contagious to students, among whom he was most popular, and his hours of clinical instruction in the wards were always attended by large numbers of them, who learned to appreciate the thoroughness of his teaching and to admire his profound knowledge of his subject.

With his literary tastes, it was inevitable that Greig Smith would be deeply interested in the project of a medical library for this district, and if, in the earlier days of the recent movement for its establishment, he did not enter into the plan with all the vigour which he was accustomed to put into any work in which he was interested, it was only because he felt that his scheme of a large and comprehensive library might be endangered by the formation of one somewhat restricted in its original outline. But when it became possible, largely, if not entirely, through his influence, to gather under one roof all the existing medical libraries of the city, his whole-hearted support of the Bristol Medical Library was of the greatest value to the profession. At the first meeting of the Library Committee in May, 1893, he was elected its Chairman, a post which he held to the time of his death. In this he was a conspicuous success, being not only able to speak with authority on the literature, both ancient and modern, of his own particular branch, but also competent from the width of his knowledge to give most useful advice about the literature of all departments of professional work. Combined with this he had an ever-watchful eye on the practical details of library work, and to all who will have to fill this position which he so adorned he set an example that will be far-reaching if not permanent in its influence for good, and which it will be difficult to equal.

Greig Smith's early training in general literature must have been particularly thorough, and this he was ever careful to foster. His omnivorous reading, which extended from the ancient classics to the latest work of fiction, gave him the power of being a most charming companion. He was a brilliant conversationalist and could talk well on an endless variety of subjects, often delighting his intimate associates with sparkling, if sometimes caustic, epigram and simile or with forcible repartee. Notwithstanding the attention he paid to literature and his own special work, he found time for indulging his intense fondness for country life, and entered into its pursuits with the same zest that he showed for all the other things to which he devoted himself. He had a small country house at Burnett, near Keynsham, in Somersetshire, to which he often betook himself when opportunity offered, and where he was always glad to see his friends. With a limited experience he had become an excellent shot, and of late years he had become passionately absorbed in golf. He was a persistent smoker and rarely missed the chance of a cigarette.

Among the recreations of his leisure time the plastic arts had a great fascination for him. Some of his figures and some medallions of his friends have great merit. In art matters generally he was an acute critic, being able to sum up, with great terseness and point, the merits and demerits of the subject under consideration. He had from time to time done many excellent pen-and-ink portraits and caricatures.

Strongly imbued with the scientific spirit and hating in his work anything that was not scrupulously exact, Greig Smith could find no atom of sympathy for that which savoured of ignorance or stupidity or carelessness, and he never hesitated, even if it caused some temporary annoyance to those from whom he differed, to expose in the most uncompromising manner any departure from what he considered to be the essential duty of all seekers after professional truth. Like most men with strong force of character, Greig Smith was not free from the defects of his qualities, and although those who had seen much of him may freely grant, in the words of him whose work he knew and loved so well, that

> "He had twa faults, or maybe three,"

yet each one of us who was acquainted with his worth will fervently add two other lines from the same verse:

> "Heav'n rest his saul, whare'er he be!
> Is th' wish o' mony mae than me."

---

### BIBLIOGRAPHY OF THE WORK OF JAMES GREIG SMITH.

Case of tumour of corpus striatum; no paralysis. *Med. Times & Gaz.*, 1878, i. 333-4.

The treatment of the pyrexia of enteric fever. *Practitioner*, 1878, xxi. 20—31.

Case of stabbing of brain and wounding of middle cerebral artery. *Med. Times & Gaz.*, 1878, ii. 378-9.

Acute miliary tuberculosis. *Tr. Bristol M.-Chir. Soc.*, 1878, i. 56—59, 1 l., 1 pl.

Hemorrhage into pons and medulla. *Ibid.*, 115—117, 1 pl.

The pathology and treatment of chronic osteo-arthritis. *Bristol Roy. Infirm. Rep.*, 1878-79, i. 81—132, 10 pl.

The mode of growth of spicular osteophytes. *Ibid.*, 133—142, 1 pl.
A complicated case of herniotomy. *Ibid.*, 239—244.
Surgical report. *Ibid.*, 245—254.
Tabular reports of important operations. *Ibid.*, 255—275.
Short notes of interesting surgical cases. *Ibid.*, 277, 291, 292, 295, 296, 297, 299, 304—308.
On the prevention of blood-poisoning in the practice of surgery. *Lancet*, 1879, ii. 256—258.
Two cases of successful operation for extrophy of the bladder by a new method. *Brit. M. J.*, 1880, i. 202.
Extrophy of the bladder. *Ibid.*, 321.
Clinical lecture on medullo-arthritis. *Lancet*, 1881, ii. 1077, 1117.
Observations on the histology of fracture repair in man. *J. Anat. & Physiol.*, 1881-82, xvi. 153—191, 2 pl.
Eleven antiseptic ovariotomies. *Brit. M. J.*, 1882, i. 815.
Remarks on the early operative treatment of strumous joint-disease. *Ibid.*, ii. 356.
Gastrostomy for malignant stricture of the œsophagus; hydatid cyst of liver. *Bristol M.-Chir. J.*, 1883, i. 122—126.
Recurrent pendulous fatty tumour of thigh. *Ibid.*, 126—128, 2 pl.
Primary scirrhus of the upper jaw in a child two years old. *Ibid.*, 128, 1 pl.
Case of very large meningocele. *Ibid.*, 129, 1 pl.
Congenital deformity of right leg and left hand. *Ibid.*, 130, 1 pl.
Removal by vagina of an ovary adherent in Douglas's pouch for severe dysmenorrhœa; cure. *Lancet*, 1883, ii. 1038. Also translated *Med. contemp.*, Lisbon, 1883, i. 419.
The fixation of movable kidney by scratching its capsule through the loin. *Ibid*, 1884, ii. 10.
Ingrowing toe-nail. *Bristol M.-Chir. J.*, 1884, ii. 99—109.
An undescribed form of stricture at the orifice of the male urethra. *Ibid.*, 154—158.
Two cases of hæmophilia. *Ibid.*, 264—269.
Intestinal obstruction. *Ibid.*, 1885, iii. 1—12.
Twenty-five cases of abdominal section in the Bristol Royal Infirmary. *Lancet*, 1885, i. 104.
Abstract from a clinical lecture on five cases of laparotomy for intestinal obstruction; the mode of operating. *Brit. M. J.*, 1885, i. 1189. Also *Maryland M. J.*, 1885, xiii. 181—183. Also *Atlanta M. & S. J.*, 1885-86, N.S. ii. 407—412.
Case of successful operation for extroversion of the bladder in a female. *Lancet*, 1885, ii. 8.
The operative treatment of acute intestinal obstruction. *Brit. M. J.*, 1885, ii. 390.
Are suppurating kidneys always removable? *Lancet*, 1885, ii. 1069.

Papillomatous cystic disease of the broad ligaments; its clinical and operative features; with three cases. *Ann. Surg.*, 1885, ii. 439—449.

Removal of the uterine appendages. *Bristol M.-Chir. J.*, 1886, iv. 1—27, 73—94.

Calculus on foreign body in bladder; perforation of bladder; no symptoms. *Ibid.*, 38—42, 1 pl.

On removal of tumours of the bladder; with four cases. *Brit. M. J.*, 1886, i. 1161.

"Diseases of the Bones," pp. 93—148, in *A Manual of Surgery*, edited by F. Treves, vol. ii. Cassell and Company. London. 1886.

Uterine appendages removed by operation. *Brit. Gynæc. J.*, 1886-87, ii. 148—150.

Case of successful removal, *per vaginam*, of a cancerous (and pregnant) uterus. *Lancet*, 1887, i. 14.

*Abdominal Surgery.* 8vo. Pp. 618. J. & A. Churchill. London. 1887.

Some interesting cases of stone in the bladder. *Bristol M.-Chir. J.*, 1887, v. 177—189, 1 pl.

Note on the action of the ureters as observed during an operation for the removal of an abdominal tumour. *J. Anat. & Physiol.*, 1887—88, xxii. 496.

*Abdominal Surgery.* 2nd ed. 8vo. Pp. 792. J. & A. Churchill. London. 1888.

*Abdominal Surgery.* 3rd ed. 8vo. Pp. 815. J. & A. Churchill. London. 1889.

The present position of abdominal surgery. *Brit. M. J.*, 1890, i. 956. *Lancet*, 1890, i. 906.

Case of removal of the vermiform appendix (during a quiescent period) for recurrent attacks of inflammation. *Lancet*, 1890, i. 956—958.

Removal of the vermiform appendix. *Ibid.*, 1038.

The radical cure of hernia; inguinal, femoral, and umbilical. *Bristol M.-Chir. J.*, 1890, viii. 1—17, 1 pl.

Two cases of acute intestinal obstruction, associated with enormous secretion of peritoneal fluid; abdominal section and recovery in each. *Brit. M. J.*, 1890, i. 883.

The surgical treatment of acute intestinal obstruction. *Ibid.*, ii. 835—837. *Lancet*, 1890, ii. 304.

Reports on surgery. *Bristol M.-Chir. J.*, 1891, ix. 35—40, 99—103.

A case in which sloughs of renal tissue were passed by the urethra. *Brit. M. J.*, 1891, i. 220.

Intestinal obstruction caused by tumour; ileostomy; disappearance of tumour; subsequent enterorrhaphy; recovery. *Lancet*, 1891, i. 646.

The treatment of hernia by median abdominal section. *Brit. M. J.*, 1891, ii. 688.

The operation of combined pylorectomy and gastro-enterostomy. *Lancet*, 1891, ii. 1070.

*Abdominal Surgery.* 4th ed. 8vo. Pp. 824. J. & A. Churchill. London. 1891.

Enterostomy in intestinal obstruction. *Brit. M. J.*, 1892, i. 554. *Lancet*, 1892, i. 582.

Evacuation and drainage of intestinal contents in obstruction of the bowels. *Internat. Clin.*, 1893, 3rd ser. i. 194—202.

Modern medical journalism. *Bristol M.-Chir. J.*, 1893, xi. 217—229.

On the (so-called) spontaneous disappearance of solid abdominal tumours; with three cases. *Med.-Chir. Tr.*, 1894, lxxvii. 139—149. Abstract in *Brit. M. J.*, 1894, i. 190, and *Lancet*, 1894, i. 207.

*Chirurgie abdominale*, ouvrage traduit avec l'autorisation de l'auteur, par le Dr. Paul Vallin; précédé d'une préface du Dr. H. Duret. 8vo. Pp. 857. G. Steinheil. Paris. 1894.

The art of the surgeon. *Brit. M. J.*, 1894, ii. 249—253. *Lancet*, 1894, ii. 245—249.

Is the apposition of peritoneum to peritoneum a surgical error? *Brit. M. J.*, 1895, i. 1.

Modification of Sir Spencer Wells's catch forceps. *Ibid.*, 655.

Note on a rare condition of the omentum. *Lancet*, 1895, ii. 331.

The parietal incision in abdominal surgery. *Ann. Surg.*, 1895, xxi. 365—388.

Some difficulties in the use of the curette. *Brit. M. J.*, 1895, ii. 16.

Cœliotomy for volvulus of the sigmoid in a man aged 85; intestinal drainage; recovery. *Ibid.*, 122—124.

The diagnosis and treatment of fractures of the upper third of the femur, including the neck. *Ibid.*, 894.

Colectomy. *Ibid.*, 965.

Extra-peritoneal closure of artificial anus and fæcal fistula. *Bristol M.-Chir. J.*, 1895, xiii. 1—7.

Unusual shape and position of tumour caused by dilated gall-bladder. *Clin. Sketches*, 1895, i. 134-5.

Resection of intestine for tumour with intussusception by means of Murphy's button. *Lancet*, 1896, i. 10—13.

*Abdominal Surgery*. 5th ed. 2 vols. 8vo. Pp. 1233. J. & A. Churchill. London. 1896.

A case of fatal hemorrhage from a stercoral ulcer of the colon complicating relief of obstruction from cancer. *Lancet*, 1896, i. 1644.

"Ovariotomy," pp. 872—911, in *A System of Gynæcology*, edited by T. Clifford Allbutt and W. S. Playfair. Macmillan and Co. London. 1896.

Was Robert Houston of Glasgow the first ovariotomist? *Practitioner*, 1897, lviii. 270—285.

The pre-diagnostic treatment of grave abdominal disease. *Treatment*, 1897, i. 25—27.

A case of intestinal obstruction through a hole in the mesentery, associated with volvulus and hemorrhage into the abdominal cavity; operation; recovery. *Brit. M. J.*, 1897, i. 1022-3.

